# Supplementary material for: Systematic assessment of obesity-related risk factors in renal cancer etiology: A longitudinal risk and Mendelian randomization analysis
Source: PLoS Med. 2026 Feb 10;23(2):e1004906. doi: 10.1371/journal.pmed.1004906 (PMC12919923; doi:10.1371/journal.pmed.1004906)
Supplement: S1 Fig — NSHDS: The Northern Sweden Health and Disease Study. GWAS: Genome-wide association study. SNPs: Single nucleotide polymorphism. Exp: exposure. Med: potential mediator. Out: outcome. MR: Mendelian Randomization. IVW: Inverse Variance Weighted. (DOCX) [file pmed.1004906.s001.docx]

Alcala, Mariosa, Jacobson, Coscia-Requena, Dimou, Franklin, Martin, Davey Smith, Gunter, Brennan, Pollak, Langdon, Johansson. Systematic assessment of obesity-related risk factors in renal cancer etiology: A longitudinal risk and Mendelian randomization analysis

**S1 Figure. Flowchart of the mediation analysis.**


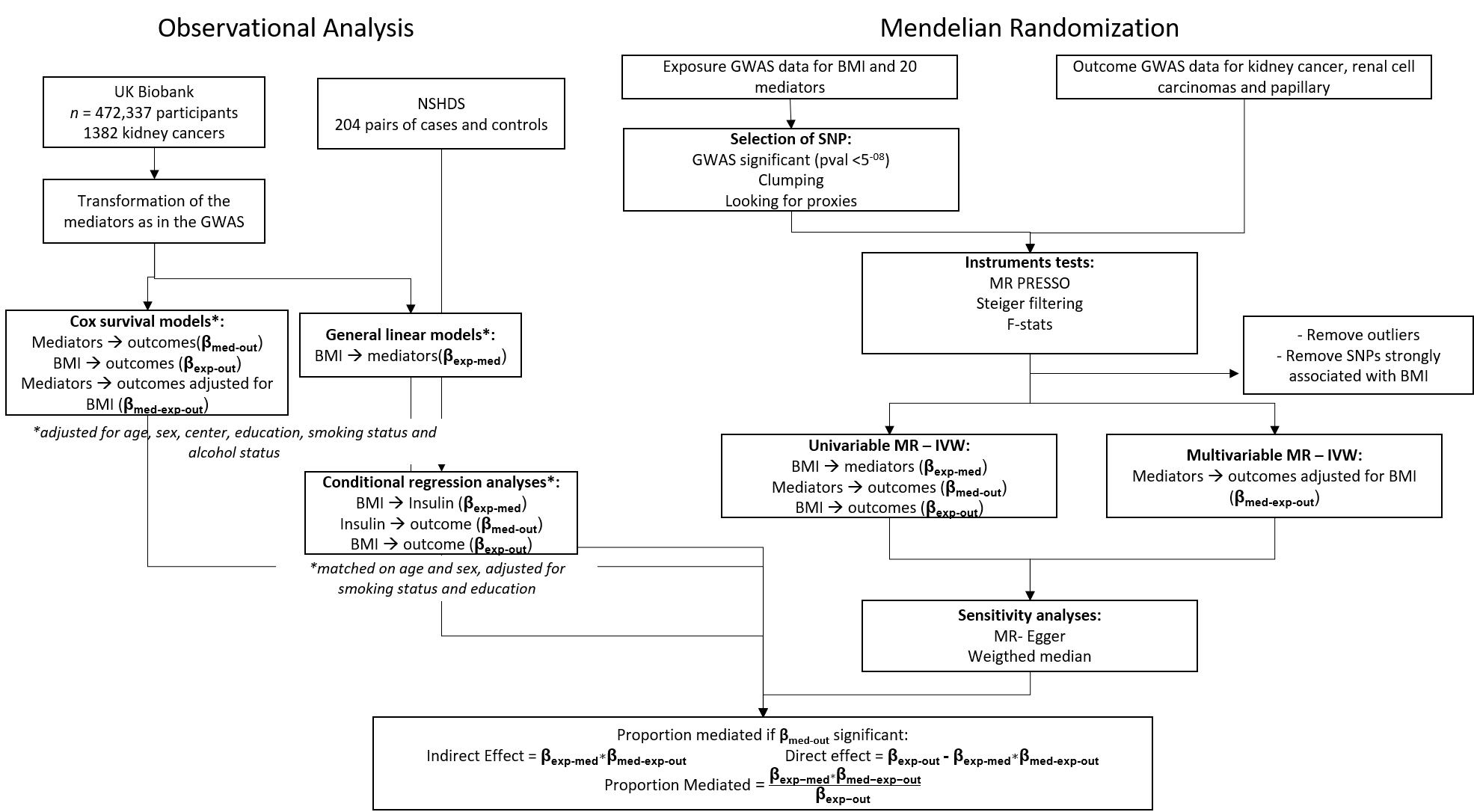


NSHDS: The Northern Sweden Health and Disease Study. GWAS: Genome-wide association study. SNPs: Single nucleotide polymorphism. Exp: exposure. Med: potential mediator. Out: outcome. MR: Mendelian Randomization. IVW: Inverse Variance Weighted.
