# Supplementary material for: Systematic assessment of obesity-related risk factors in renal cancer etiology: A longitudinal risk and Mendelian randomization analysis
Source: PLoS Med. 2026 Feb 10;23(2):e1004906. doi: 10.1371/journal.pmed.1004906 (PMC12919923; doi:10.1371/journal.pmed.1004906)
Supplement: S2 Fig — Indirect effect estimates for BMI through each risk factor with renal cancer risk as calculated with the product method, confidence intervals estimated through the Sobel method. MR: Mendelian Randomization. BP: Blood pressure. HDL: High-density lipoprotein. SHBG: Sex-hormone binding globulin. CI: Confidence Interval. (DOCX) [file pmed.1004906.s002.docx]

Alcala, Mariosa, Jacobson, Coscia-Requena, Dimou, Franklin, Martin, Davey Smith, Gunter, Brennan, Pollak, Langdon, Johansson. Systematic assessment of obesity-related risk factors in renal cancer etiology: A longitudinal risk and Mendelian randomization analysis

**S2 Figure. Indirect effect estimates for BMI in the mediation analysis.**


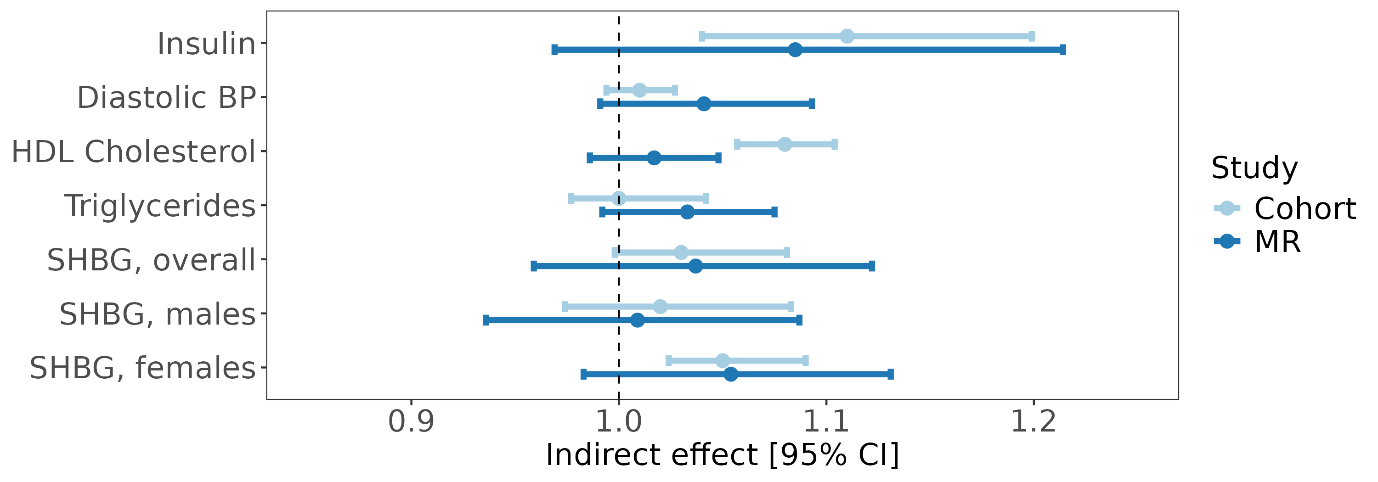


Indirect effect estimates for BMI through each risk factor with renal cancer risk as calculated with the product method, confidence intervals estimated through the Sobel method. MR: Mendelian Randomization. BP: Blood pressure. HDL: High-density lipoprotein. SHBG: Sex-hormone binding globulin. CI: Confidence Interval.
