# Supplementary material for: Systematic assessment of obesity-related risk factors in renal cancer etiology: A longitudinal risk and Mendelian randomization analysis
Source: PLoS Med. 2026 Feb 10;23(2):e1004906. doi: 10.1371/journal.pmed.1004906 (PMC12919923; doi:10.1371/journal.pmed.1004906)
Supplement: S1 Text — Table A. Potential obesity-related risk factors for renal cell carcinomas, in the Mendelian Randomization analyses. *Removed due to missingness in the outcome SNPs, outliers identified by MR-PRESSO or removed by steiger filtering. SNP: single-nucleotide polymorphism. GWAS: Genome-wide association study. INTR: Inverse normal transformation of rank. HbA1c: glycated hemoglobin. IGF-1: Insulin-like growth factor-1. eGFR: estimated glomerular filtration rate. HDL/LDL: High/Low-density lipoproteins. SHBG: Sex-hormone binding globulin. Table B. Potential obesity-related risk factors for clear cell renal cell carcinomas, in the Mendelian randomization analyses. *Removed due to missingness in the outcome SNPs, outliers identified by MR-PRESSO or removed by steiger filtering. SNP: single-nucleotide polymorphism. GWAS: Genome-wide association study. INTR: Inverse normal transformation of rank. HbA1c: glycated hemoglobin. IGF-1: Insulin-like growth factor-1. eGFR: estimated glomerular filtration rate. HDL/LDL: High/Low-density lipoproteins. SHBG: Sex-hormone binding globulin. Table C. Potential obesity-related risk factors for papillary renal cell carcinoma, in the Mendelian Randomization analysis. *Removed due to missingness in the outcome SNPs, outliers identified by MR-PRESSO or removed by steiger filtering. SNP: single-nucleotide polymorphism. GWAS: Genome-wide association study. INTR: Inverse normal transformation of rank. HbA1c: glycated hemoglobin. IGF-1: Insulin-like growth factor-1. eGFR: estimated glomerular filtration rate. HDL/LDL: High/Low-density lipoproteins. SHBG: Sex-hormone binding globulin. Table D. Characteristics of the UKB population, by renal cell carcinomas subtypes. 1n (%); Median (IQR). RCC: Renal cell carcinoma. ccRCC: clear cell renal cell carcinoma. pRCC: papillary renal cell carcinoma. HbA1c: glycated hemoglobin. IGF-1: Insulin-like growth factor-1. eGFR: estimated glomerular filtration rate. HDL/LDL: High/Low-density lipoproteins. SHBG: Sex-hormon [file pmed.1004906.s003.docx]

Alcala, Mariosa, Jacobson, Coscia-Requena, Dimou, Franklin, Martin, Davey Smith, Gunter, Brennan, Pollak, Langdon, Johansson. Systematic assessment of obesity-related risk factors in renal cancer etiology: A longitudinal risk and Mendelian randomization analysis

Contents

[Abbreviations 2](#_Toc219794063)

[Table A. Potential obesity-related risk factors for renal cell carcinomas, in the Mendelian Randomization analyses. 3](#_Toc219794064)

[Table B. Potential obesity-related risk factors for clear cell renal cell carcinomas, in the Mendelian Randomization analyses. 4](#_Toc219794065)

[Table C. Potential obesity-related risk factors for papillary renal cell carcinoma, in the Mendelian Randomization analysis. 5](#_Toc219794066)

[Table D. Characteristics of the UKB population, by renal cell carcinomas subtypes 6](#_Toc219794067)

[Table E. Characteristics of the UKB population, by sex 7](#_Toc219794068)

[Table F. Characteristics of the Northern Sweden Health and Disease Study (NSHDS) population 8](#_Toc219794069)

[Table G. Association of BMI with each obesity-related risk factor 9](#_Toc219794070)

[Table H. Association of potential obesity-related risk factors with risk of renal cell carcinoma using Mendelian randomization and prospective cohort analyses. 10](#_Toc219794071)

[Table I. Association of potential obesity-related risk factors with risk of clear cell renal cell carcinoma using Mendelian randomization and prospective cohort analyses. 11](#_Toc219794072)

[Table J. Association of potential obesity-related risk factors with risk of papillary renal cell carcinoma using Mendelian randomization and prospective cohort analyses. 12](#_Toc219794073)

[Table K. Comparison between the adjusted and the non-adjusted model for specific female factors. 13](#_Toc219794074)

[Table L. Beta estimates between potential mediators 14](#_Toc219794075)

[Table M. Proportion of BMI effect on renal cell carcinoma mediated. 15](#_Toc219794076)

[Table N. Proportion of BMI effect on ccRCC mediated. 15](#_Toc219794077)

[Table O. Multivariable analyses of mediators on renal cell carcinomas. 16](#_Toc219794078)

# Abbreviations

BMI: Body mass index

DBP: Diastolic blood pressure

eGFR: Estimated glomerular filtration rate

GWAS: Genome wide association study

HbA1c: Glycated hemoglobin

HDL: High-density lipoprotein

LDL: Low-density lipoprotein

MR: Mendelian Randomization

NSHDS: The Northern Sweden Health and Disease Study

RCC: Renal cell carcinoma

SBP: Systolic blood pressure

SHBG: Sex-hormone binding globulin

SNP: Single nucleotide polymorphism

UKB: UK Biobank

## Table A. Potential obesity-related risk factors for renal cell carcinomas, in the Mendelian Randomization analyses.

|  |  |  |  |  | **All selected SNPs** | | **Final analyses** | | | |
| --- | --- | --- | --- | --- | --- | --- | --- | --- | --- | --- |
| **Risk factors** | **Transformation** | **Publication** | **Sample source** | **Sample size** | **N SNPs** | **Variance Explained (%)** | **N SNPs removed*** | **N SNPs** | **Variance Explained (%)** | **F-statistic** |
| BMI | Standardized | Yengo, 2018[1] | UK Biobank & 114 joint GWAS | 690,800 | 956 | 7.64 | 13 | 943 | 7.78 | 58.2 |
| Fasting insulin | Natural log | Lagou, 2021[2] | 38 cohorts | 93,170 | 12 | 0.55 | 1 | 11 | 0.48 | 41.2 |
| Glucose | Natural log | Internal GWAS | UK Biobank | 327,249 | 92 | 2.39 | 2 | 90 | 2.43 | 85.6 |
| HbA1c | Natural log | Internal GWAS | UK Biobank | 357,630 | 334 | 8.06 | 6 | 328 | 8.14 | 86.5 |
| Diastolic blood pressure | Z-score | Johansson, 2019[3] | UK Biobank | 375,091 | 260 | 3.45 | 8 | 252 | 3.37 | 49.6 |
| Systolic blood pressure | Z-score | Johansson, 2019[3] | UK Biobank | 375,091 | 222 | 2.94 | 5 | 217 | 2.92 | 50.0 |
| eGFR | INTR and Z-score | Karczewski, 2025[4] | UK Biobank | 401,867 | 774 | 13.8 | 34 | 740 | 12.8 | 69.2 |
| IGF-1 | Natural log | Sinnott-Armstrong,2021[5] | UK Biobank | 342,439 | 557 | 12.9 | 9 | 548 | 12.8 | 79.3 |
| **Lipids** |  |  |  |  |  |  |  |  |  |  |
| Total cholesterol | Standardized | Graham, 2021[6] | Global Lipids Genetics consortium | 925,143 | 726 | 10.5 | 10 | 716 | 10.4 | 132 |
| HDL | Standardized | Graham, 2021[6] |  | 882,706 | 835 | 11.5 | 22 | 813 | 11.2 | 120 |
| *small* | INTR and Z-score | Davyson, 2023[7] | UK Biobank | 88,329 | 39 | 4.30 | 0 | 39 | 4.30 | 97.7 |
| *medium* | INTR and Z-score | Davyson, 2023[7] | UK Biobank | 88,329 | 91 | 9.45 | 1 | 90 | 9.37 | 92.3 |
| *large* | INTR and Z-score | Davyson, 2023[7] | UK Biobank | 88,329 | 107 | 13.1 | 0 | 107 | 13.1 | 109 |
| *very large* | INTR and Z-score | Davyson, 2023[7] | UK Biobank | 88,329 | 95 | 13.2 | 1 | 94 | 13.0 | 123 |
| LDL | Standardized | Graham, 2021[6] | Global Lipids Genetics consortium | 837,495 | 559 | 10.2 | 7 | 552 | 10.1 | 150 |
| Triglycerides | Natural log | Graham, 2021[6] |  | 857,850 | 668 | 8.65 | 16 | 652 | 8.57 | 110 |
| **Sex hormones** |  |  |  |  |  |  |  |  |  |  |
| SHBG | Natural log | Ruth, 2020[8] | UK Biobank | 370,125 | 711 | 19.3 | 18 | 693 | 19.4 | 101 |
| *male* | Natural log | Ruth, 2020 [8] | UK Biobank | 180,726 | 376 | 20.8 | 11 | 365 | 20.5 | 101 |
| *female* | INTR and Z-score | Ruth, 2020 [8] | UK Biobank | 189,473 | 351 | 16.3 | 12 | 339 | 16.0 | 89.1 |
| Total testosterone |  |  |  |  |  |  |  |  |  |  |
| *male* | INTR and Z-score | Ruth, 2020 [8] | UK Biobank | 194,453 | 237 | 10.3 | 8 | 229 | 10.68 | 85.5 |
| *female* | Natural log | Ruth, 2020 [8] | UK Biobank | 230,454 | 286 | 9.46 | 7 | 279 | 9.41 | 76.4 |

*Removed due to missingness in the outcome SNPs, outliers identified by MR-PRESSO or removed by steiger filtering. SNP: single-nucleotide polymorphism. GWAS: Genome-wide association study. INTR: Inverse normal transformation of rank. HbA1c: glycated hemoglobin. IGF-1: Insulin-like growth factor-1. eGFR: estimated glomerular filtration rate. HDL/LDL: High/Low-density lipoproteins. SHBG: Sex-hormone binding globulin.

## Table B. Potential obesity-related risk factors for clear cell renal cell carcinomas, in the Mendelian Randomization analyses.

|  |  |  |  |  | **All selected SNPs** | | **Final analyses** | | | |
| --- | --- | --- | --- | --- | --- | --- | --- | --- | --- | --- |
| **Risk factors** | **Transformation** | **Publication** | **Sample source** | **Sample size** | **N SNPs** | **Variance Explained (%)** | **N SNPs removed*** | **N SNPs** | **Variance Explained (%)** | **F-statistic** |
| BMI | Standardized | Yengo, 2018[1] | UK Biobank& 114 joint GWAS | 690,800 | 956 | 7.64 | 10 | 946 | 7.77 | 57.9 |
| Fasting insulin | Natural log | Lagou, 2021[2] | 38 cohorts | 93,170 | 12 | 0.55 | 1 | 11 | 0.48 | 41.2 |
| Glucose | Natural log | Internal GWAS | UK Biobank | 327,249 | 90 | 2.37 | 1 | 89 | 2.43 | 86.6 |
| HbA1c | Natural log | Internal GWAS | UK Biobank | 357,630 | 331 | 7.95 | 4 | 327 | 8.07 | 86.3 |
| Diastolic blood pressure | Z-score | Johansson, 2019[3] | UK Biobank | 375,091 | 259 | 3.44 | 6 | 253 | 3.39 | 49.7 |
| Systolic blood pressure | Z-score | Johansson, 2019[3] | UK Biobank | 375,091 | 223 | 2.96 | 2 | 221 | 2.95 | 49.9 |
| eGFR | INTR and Z-score | Karczewski, 2025[4] | UK Biobank | 401,867 | 756 | 13.5 | 26 | 730 | 13.2 | 71.8 |
| IGF-1 | Natural log | Sinnott-Armstrong,2021[5] | UK Biobank | 342,439 | 552 | 12.8 | 7 | 545 | 12.8 | 79.5 |
| **Lipids** |  |  |  |  |  |  |  |  |  |  |
| Total cholesterol | Standardized | Graham, 2021[6] | Global Lipids Genetics consortium | 925,143 | 720 | 10.4 | 7 | 713 | 10.3 | 132 |
| HDL | Standardized | Graham, 2021[6] |  | 882,706 | 830 | 11.4 | 17 | 813 | 11.2 | 120 |
| *small* | INTR and Z-score | Davyson, 2023[7] | UK Biobank | 88,329 | 37 | 4.20 | 0 | 37 | 4.20 | 101 |
| *medium* | INTR and Z-score | Davyson, 2023[7] | UK Biobank | 88,329 | 90 | 9.41 | 1 | 89 | 9.33 | 93.0 |
| *large* | INTR and Z-score | Davyson, 2023[7] | UK Biobank | 88,329 | 104 | 13.0 | 2 | 102 | 12.9 | 112 |
| *very large* | INTR and Z-score | Davyson, 2023[7] | UK Biobank | 88,329 | 93 | 13.1 | 2 | 91 | 13.0 | 127 |
| LDL | Standardized | Graham, 2021[6] | Global Lipids Genetics consortium | 837,495 | 557 | 10.1 | 5 | 552 | 10.0 | 149 |
| Triglycerides | Natural log | Graham, 2021[6] |  | 857,850 | 665 | 8.61 | 12 | 653 | 8.55 | 110 |
| **Sex hormones** |  |  |  |  |  |  |  |  |  |  |
| SHBG | Natural log | Ruth, 2020[8] | UK Biobank | 370,125 | 691 | 18.7 | 18 | 673 | 18.7 | 100 |
| *male* | Natural log | Ruth, 2020 [8] | UK Biobank | 180,726 | 364 | 20.5 | 15 | 349 | 20.0 | 103 |
| *female* | INTR and Z-score | Ruth, 2020 [8] | UK Biobank | 189,473 | 349 | 16.3 | 12 | 337 | 15.9 | 89.2 |
| Total testosterone |  |  |  |  |  |  |  |  |  |  |
| *male* | INTR and Z-score | Ruth, 2020 [8] | UK Biobank | 194,453 | 231 | 9.89 | 8 | 223 | 10.2 | 84.2 |
| *female* | INTR and Z-score | Ruth, 2020 [8] | UK Biobank | 230,454 | 281 | 9.33 | 6 | 275 | 9.34 | 77.3 |

*Removed due to missingness in the outcome SNPs, outliers identified by MR-PRESSO or removed by steiger filtering. SNP: single-nucleotide polymorphism. GWAS: Genome-wide association study. INTR: Inverse normal transformation of rank. HbA1c: glycated hemoglobin. IGF-1: Insulin-like growth factor-1. eGFR: estimated glomerular filtration rate. HDL/LDL: High/Low-density lipoproteins. SHBG: Sex-hormone binding globulin.

## Table C. Potential obesity-related risk factors for papillary renal cell carcinoma, in the Mendelian Randomization analysis.

|  |  |  |  |  | **All selected SNPs** | | **Final analyses** | | | |
| --- | --- | --- | --- | --- | --- | --- | --- | --- | --- | --- |
| **Risk factors** | **Transformation** | **Publication** | **Sample source** | **Sample size** | **N SNPs** | **Variance Explained (%)** | **N SNPs removed*** | **N SNPs** | **Variance Explained (%)** | **F-statistic** |
| BMI | Standardized | Yengo, 2018[1] | UK Biobank & 114 joint GWAS | 690,800 | 954 | 7.63 | 1 | 953 | 7.63 | 58.2 |
| Fasting insulin | Natural log | Lagou, 2021[2] | 38 cohorts | 93,170 | 12 | 0.55 | 0 | 12 | 0.55 | 43.1 |
| Glucose | Natural log | Internal GWAS | UK Biobank | 327,249 | 90 | 2.37 | 0 | 90 | 2.37 | 86.2 |
| HbA1c | Natural log | Internal GWAS | UK Biobank | 357,630 | 329 | 7.92 | 1 | 328 | 7.91 | 85.4 |
| Diastolic blood pressure | Z-score | Johansson, 2019[3] | UK Biobank | 375,091 | 259 | 3.44 | 1 | 258 | 3.47 | 49.8 |
| Systolic blood pressure | Z-score | Johansson, 2019[3] | UK Biobank | 375,091 | 223 | 2.96 | 1 | 222 | 2.95 | 49.9 |
| eGFR | INTR and Z-score | Karczewski, 2025[4] | UK Biobank | 401,867 | 755 | 13.5 | 7 | 748 | 13.3 | 70.8 |
| IGF-1 | Natural log | Sinnott-Armstrong,2021[5] | UK Biobank | 342,439 | 552 | 12.8 | 0 | 552 | 12.8 | 79.4 |
| **Lipids** |  |  |  |  |  |  |  |  |  |  |
| Total cholesterol | Standardized | Graham, 2021[6] | Global Lipids Genetics consortium | 925,143 | 720 | 10.4 | 2 | 718 | 10.4 | 132 |
| HDL | Standardized | Graham, 2021[6] |  | 882,706 | 830 | 11.4 | 2 | 828 | 11.4 | 121 |
| *small* | INTR and Z-score | Davyson, 2023[7] | UK Biobank | 88,329 | 37 | 4.20 | 0 | 37 | 4.20 | 101 |
| *medium* | INTR and Z-score | Davyson, 2023[7] | UK Biobank | 88,329 | 90 | 9.41 | 0 | 90 | 9.41 | 92.7 |
| *large* | INTR and Z-score | Davyson, 2023[7] | UK Biobank | 88,329 | 104 | 13.0 | 0 | 104 | 13.0 | 111 |
| *very large* | INTR and Z-score | Davyson, 2023[7] | UK Biobank | 88,329 | 93 | 13.1 | 0 | 93 | 13.1 | 125 |
| LDL | Standardized | Graham, 2021[6] | Global Lipids Genetics consortium | 837,495 | 557 | 10.1 | 1 | 556 | 10.1 | 150 |
| Triglycerides | Natural log | Graham, 2021[6] |  | 857,850 | 665 | 8.61 | 2 | 663 | 8.61 | 109 |
| **Sex hormones** |  |  |  |  |  |  |  |  |  |  |
| SHBG | Natural log | Ruth, 2020[8] | UK Biobank | 370,125 | 687 | 18.7 | 3 | 684 | 19.1 | 101 |
| *male* | Natural log | Ruth, 2020 [8] | UK Biobank | 180,726 | 362 | 20.5 | 5 | 357 | 20.5 | 103 |
| *female* | INTR and Z-score | Ruth, 2020 [8] | UK Biobank | 189,473 | 348 | 16.3 | 0 | 348 | 16.3 | 88.7 |
| Total testosterone |  |  |  |  |  |  |  |  |  |  |
| *male* | INTR and Z-score | Ruth, 2020 [8] | UK Biobank | 194,453 | 230 | 9.87 | 0 | 230 | 9.87 | 83.6 |
| *female* | Natural log | Ruth, 2020 [8] | UK Biobank | 230,454 | 281 | 9.33 | 1 | 280 | 9.32 | 76.8 |

*Removed due to missingness in the outcome SNPs, outliers identified by MR-PRESSO or removed by steiger filtering. SNP: single-nucleotide polymorphism. GWAS: Genome-wide association study. INTR: Inverse normal transformation of rank. HbA1c: glycated hemoglobin. IGF-1: Insulin-like growth factor-1. eGFR: estimated glomerular filtration rate. HDL/LDL: High/Low-density lipoproteins. SHBG: Sex-hormone binding globulin.

## Table D. Characteristics of the UKB population, by renal cell carcinomas subtypes

|  | **Overall**  **N = 472,337^1^** | **Participants diagnosed with a RCC**  **N = 1,382^1^** | **Participants diagnosed with ccRCC**  **N = 1,258^1^** | **Participants diagnosed with a pRCC**  **N = 80^1^** |
| --- | --- | --- | --- | --- |
| Person-years | 5,586,414 | 9148 | 8307 | 537 |
| Females | 254,874 (54%) | 496 (36%) | 455 (36%) | 18 (22%) |
| Age at enrolment (yrs) | 58 (50, 63) | 62 (56, 66) | 61 (56, 66) | 63 (58, 66) |
| BMI (kg/m2) | 26.7 (24.1, 29.9) | 28.3 (25.5, 31.7) | 28.4 (25.6, 31.7) | 27.6 (24.9, 30.5) |
| Smoking status |  |  |  |  |
| Never | 258,301 (55%) | 601 (44%) | 557 (45%) | 36 (46%) |
| Former | 161,556 (34%) | 576 (42%) | 524 (42%) | 34 (43%) |
| Current | 50,112 (11%) | 197 (14%) | 170 (14%) | 9 (11%) |
| Alcohol status |  |  |  |  |
| Never | 20,810 (4.4%) | 42 (3.1%) | 36 (2.9%) | 1 (1.30%) |
| Former | 16,705 (3.5%) | 67 (4.9%) | 60 (4.8%) | 4 (5.10%) |
| Current | 433,639 (92%) | 1,265 (92%) | 1,154 (92%) | 73 (94.0%) |
| Glucose (mmol/L) | 4.93 (4.60, 5.31) | 5.01 (4.67, 5.44) | 5.01 (4.67, 5.44) | 5.02 (4.71, 5.39) |
| HbA1c (mmol/mol) | 35.2 (32.7, 37.9) | 36.1 (33.5, 39.1) | 36.0 (33.5, 39.1) | 36.3 (34.0, 39.1) |
| Diastolic blood pressure (mmHg) | 82 (75, 88) | 84 (77, 90) | 83 (77, 90) | 81 (76, 91) |
| Systolic blood pressure (mmHg) | 136 (124, 148) | 142 (130, 154) | 142 (129, 154) | 140 (127, 154) |
| Estimated glomerular filtration rate (eGFR, mL/min/1.73 m) | 97 (87, 104) | 94 (81, 100) | 94 (81, 100) | 90 (80, 98) |
| Total cholesterol (mmol/L) | 4.60 (3.99, 5.23) | 4.33 (3.67, 4.98) | 4.34 (3.70, 4.98) | 4.24 (3.68, 4.89) |
|  |  |  |  |  |
| HDL (nmol/L) | 1.40 (1.17, 1.67) | 1.25 (1.05, 1.51) | 1.25 (1.05, 1.51) | 1.27 (1.10, 1.54) |
| Small particles (mmol/L) | 0.01 (0.009, 0.011) | 0.0095 (0.0086, 0.0104) | 0.01 (0.009, 0.011) | 0.0094 (0.0084, 0.0101) |
| Medium particles (mmol/L) | 0.004 (0.003, 0.005) | 0.0035 (0.0030, 0.0042) | 0.004 (0.003, 0.004) | 0.0035 (0.0030, 0.0041) |
| Large particles (mmol/L) | 0.001 (0.001, 0.002) | 0.0010 (0.0006, 0.0015) | 0.001 (0.001, 0.002) | 0.0009 (0.0007, 0.0016) |
| Very large particles (mmol/L) | 0.0002 (0.0002, 0.0003) | 0.0002 (0.0001, 0.0002) | 0.0002 (0.0001, 0.0002) | 0.0002 (0.0001, 0.0003) |
| LDL (nmol/L) | 3.52 (2.95, 4.12) | 3.39 (2.79, 4.03) | 3.39 (2.79, 4.03) | 3.22 (2.76, 3.77) |
| Triglycerides (nmol/L) | 1.48 (1.04, 2.14) | 1.69 (1.18, 2.44) | 1.68 (1.18, 2.43) | 1.44 (0.92, 2.18) |
| SHBG (mmol/L) | 45 (32, 64) | 40 (30, 55) | 40 (30, 54) | 42 (32, 58) |
| Males | 37 (28, 48) | 36 (28, 48) | 36 (28, 48) | 41 (32, 53) |
| Females | 56 (40, 77) | 48 (35, 67) | 47 (35, 67) | 56 (38, 106) |
| Testosterone (mmol/L) |  |  |  |  |
| Males | 11.6 (9.5, 14.2) | 11.1 (8.90, 13.5) | 11.1 (8.9, 13.5) | 11.6 (9.00, 13.7) |
| Females | 1.02 (0.72, 1.38) | 1.03 (0.73, 1.41) | 1.04 (0.73, 1.41) | 0.75 (0.59, 1.18) |

^1^n (%); Median (IQR). RCC: Renal cell carcinoma. ccRCC: clear cell renal cell carcinoma. pRCC: papillary renal cell carcinoma. HbA1c: glycated hemoglobin. IGF-1: Insulin-like growth factor-1. eGFR: estimated glomerular filtration rate. HDL/LDL: High/Low-density lipoproteins. SHBG: Sex-hormone binding globulin.

## Table E. Characteristics of the UKB population, by sex

|  | | **Females**  **N = 254,874^1^** | **Males**  **N = 217,463^1^** |
| --- | --- | --- | --- |
| BMI (kg/m2) | | 26.1 (23.4, 29.7) | 27.3 (25.0, 30.1) |
| Waist-to-hip ratio | | 0.81 (0.77, 0.86) | 0.93 (0.89, 0.98) |
| Smoking status | |  |  |
| Never | | 151,857 (60%) | 106,444 (49%) |
| Former | | 78,997 (31%) | 82,559 (38%) |
| Current | | 22,786 (9.0%) | 27,326 (13%) |
| Alcohol status | |  |  |
| Never | | 14,805 (5.8%) | 6,005 (2.8%) |
| Former | | 9,160 (3.6%) | 7,545 (3.5%) |
| Current | | 230,301 (91%) | 203,338 (94%) |
| Glucose (mmol/L) | 4.91 (4.59, 5.27) | | 4.95 (4.60, 5.35) |
| HbA1c (mmol/mol) | 35.1 (32.7, 37.7) | | 35.3 (32.8, 38.1) |
| Diastolic blood pressure (mmHg) | 80 (74, 87) | | 84 (77, 91) |
| Systolic blood pressure (mmHg) | | 133 (121, 147) | 139 (129, 151) |
| eGFR (mL/min/1.73 m) | | 97 (87, 104) | 97 (87, 103) |
| Total cholesterol (mmol/L) | | 4.80 (4.20, 5.42) | 4.37 (3.77, 4.98) |
| HDL (nmol/L) | | 1.55 (1.32, 1.82) | 1.24 (1.06, 1.45) |
| LDL (nmol/L) | | 3.56 (3.00, 4.17) | 3.46 (2.87, 4.05) |
| Triglycerides (nmol/L) | | 1.33 (0.96, 1.88) | 1.69 (1.18, 2.45) |
| SHBG (mmol/L) | | 56 (40, 77) | 37 (28, 48) |
| Testosterone (mmol/L) | | 1.02 (0.72, 1.38) | 11.6 (9.5, 14.2) |

^1^n (%); Median (IQR). HbA1c: glycated hemoglobin. IGF-1: Insulin-like growth factor-1.

eGFR: estimated glomerular filtration rate. HDL/LDL: High/Low-density lipoproteins.

SHBG: Sex-hormone binding globulin.

## Table F. Characteristics of the Northern Sweden Health and Disease Study (NSHDS) population

|  | **Cases**  **N=204^1^** | **Controls**  **N=204^1^** |
| --- | --- | --- |
| Females | 85 (42%) | 86 (42%) |
| Age at enrollment (years) | 52.9 (51.9, 54.0) | 52.9 (51.8, 53.9) |
| BMI (kg/m2) | 27.2 (26.6, 27.8) | 26.5 (25.9, 27.0) |
| Missing | 4 (2%) | 2 (1%) |
| Smoking status |  |  |
| Never | 104 (51%) | 111 (54%) |
| Former | 60 (29%) | 66 (32%) |
| Current | 32 (16%) | 26 (13%) |
| Missing | 8 (4%) | 3 (1%) |
| Alcohol (g/day) | 4.38 (3.56, 5.19) | 4.46 (3.28, 5.64) |
| Diabetes |  |  |
| Yes | 5 (2.5%) | 2 (0.98%) |
| Missing | 6 (2.9%) | 1 (0.49%) |
| Insulin (mU/L) | 7.84 (6.98, 8.69) | 7.17 (5.98, 8.35) |

**^1^**n (%); Mean (95% confidence interval)

## Table G. Association of BMI with each obesity-related risk factor

|  | **Mendelian Randomization (MR)** | | | **Prospective cohort analyses** | | |
| --- | --- | --- | --- | --- | --- | --- |
| **Risk factor** | **SNPs** | **beta (95%CI) IVW** | **P-value** | **N** | **beta (95%CI)** | **P-value** |
| Fasting insulin | 753 | 0.16 [0.14, 0.17] | < 2 x10^-16^ | 485 | 0.35 [0.30, 0.40] | < 2 x10^-16^ |
| Glucose* | 940 | 0.03 [0.03, 0.03] | < 2 x10^-16^ | 402,719 | 0.03 [0.03, 0.03] | < 2 x10^-16^ |
| HbA1c* | 912 | 0.04 [0.03, 0.04] | < 2 x10^-16^ | 437,211 | 0.04 [0.04, 0.04] | < 2 x10^-16^ |
| Diastolic blood pressure*** | 934 | 0.17 [0.15, 0.19] | < 2 x10^-16^ | 444,311 | 0.27 [0.27, 0.27] | < 2 x10^-16^ |
| Systolic blood pressure*** | 939 | 0.09 [0.07, 0.11] | < 2 x10^-16^ | 449,141 | 0.16 [0.16, 0.16] | < 2 x10^-16^ |
| eGFR** | 915 | -0.04 [-0.06, -0.02] | < 2 x10^-16^ | 439,977 | -0.07 [-0.07, -0.07] | < 2 x10^-16^ |
| IGF-1* | 910 | -0.13 [-0.26, 0.00] | 0.044 | 437,807 | -0.04 [-0.04,-0.04] | < 2 x10^-16^ |
| **Lipids** |  |  |  |  |  |  |
| Total cholesterol*†* | 934 | -0.01 [-0.03, 0.00] | 0.071 | 440,187 | -0.03 [-0.03,-0.03] | < 2 x10^-16^ |
| HDL*†* | 929 | -0.26 [-0.28,-0.24] | < 2 x10^-16^ | 403,001 | -0.31 [-0.32,-0.31] | < 2 x10^-16^ |
| *Small*** | 895 | -0.02 [-0.04, 0.01] | 0.252 | 257,322 | 0.06 [ 0.06, 0.07] | < 2 x10^-16^ |
| *Medium*** | 881 | -0.19 [-0.22,-0.17] | < 2 x10^-16^ | 257,322 | -0.18 [-0.18,-0.18] | < 2 x10^-16^ |
| *Large*** | 822 | -0.28 [-0.30,-0.25] | < 2 x10^-16^ | 257,322 | -0.33 [-0.33,-0.33] | < 2 x10^-16^ |
| *Very large*** | 827 | -0.28 [-0.30,-0.25] | < 2 x10^-16^ | 257,322 | -0.32 [-0.32,-0.31] | < 2 x10^-16^ |
| LDL*†* | 938 | 0.00 [-0.01, 0.02] | 0.978 | 439,367 | 0.03 [0.03, 0.03] | < 2 x10^-16^ |
| Triglycerides* | 922 | 0.21 [0.20, 0.23] | < 2 x10^-16^ | 439,843 | 0.16 [0.16, 0.16] | < 2 x10^-16^ |
| **Sex-hormones** |  |  |  |  |  |  |
| SHBG* | 885 | -0.13 [-0.14,-0.12] | < 2 x10^-16^ | 399,203 | -0.18 [-0.18,-0.18] | < 2 x10^-16^ |
| *Male** | 871 | -0.09 [-0.10,-0.08] | < 2 x10^-16^ | 186,319 | -0.14 [-0.15,-0.14] | < 2 x10^-16^ |
| *Female*** | 842 | -0.15 [-0.16,-0.14] | < 2 x10^-16^ | 212,884 | -0.40 [-0.41,-0.40] | < 2 x10^-16^ |
| Total testosterone |  |  |  |  |  |  |
| *Male*** | 887 | -0.25 [-0.27,-0.23] | < 2 x10^-16^ | 201,507 | -0.35 [-0.36,-0.35] | < 2 x10^-16^ |
| *Female** | 914 | 0.08 [0.06, 0.10] | < 2 x10^-16^ | 197,957 | 0.04 [0.04, 0.04] | < 2 x10^-16^ |

Each cox proportional hazard model was adjusted for age, sex, center of recruitment, education, smoking and alcohol drinking status. *log-transformed, **inverse-normal transformation of rank and Z-score, ***Z-score transformation, †standardized and 1-unit increase in Estradiol. Fasting insulin association with BMI was assess in NSHDS. HbA1c: glycated hemoglobin. IGF-1: Insulin-like growth factor-1. eGFR: estimated glomerular filtration rate. HDL/LDL: High/Low-density lipoproteins. SHBG: Sex-hormone binding globulin.

## Table H. Association of potential obesity-related risk factors with risk of renal cell carcinoma using Mendelian randomization and prospective cohort analyses.

|  | **Mendelian Randomization (MR)** | | | | | | | **Prospective cohort analyses** | | |
| --- | --- | --- | --- | --- | --- | --- | --- | --- | --- | --- |
| **Risk factor** | **SNPs** | **OR (95%CI) IVW** | **MR-Egger intercept**  **(p-value)** | **OR Weighted median** | **SNPs removed⬧** | **Corrected OR (95%CI) IVW** | **Corrected MR-Egger intercept**  **(p-value)** | **N** | **N cases** | **HR/OR (95%CI)** |
| BMI† | 956 | 1.45 [1.35,1.55] | 0.07 | 1.47 [1.34, 1.61] | 13 | 1.44 [1.35,1.53] | 0.10 | 461,156 | 1345 | 1.33 [1.26,1.39] |
| Fasting insulinª | 12 | 1.72 [0.84,3.52] | 0.14 | 2.36 [1.21, 4.62] | 1 | 2.24 [1.19,4.22] | 0.18 | 378 | 189 | 1.43 [1.02, 2.00] |
| Glucose* | 92 | 0.39 [0.17, 0.87] | 0.74 | 0.62 [0.24, 1.56] | 2 | 0.38 [0.18, 0.80] | 0.88 | 398,602 | 1142 | 1.64 [1.24, 2.17] |
| HbA1c* | 334 | 0.76 [0.46, 1.25] | 0.85 | 0.83 [0.43, 1.60] | 6 | 0.74 [0.46, 1.18] | 0.57 | 432,802 | 1269 | 1.87 [1.35, 2.29] |
| Diastolic blood pressure*** | 260 | 1.20 [1.07,1.34] | 0.39 | 1.09 [0.96, 1.24] | 8 | 1.14 [1.04,1.26] | 0.73 | 436,517 | 1271 | 1.11 [1.05,1.17] |
| Systolic blood pressure*** | 222 | 1.03 [0.91,1.16] | 0.45 | 1.02 [0.88, 1.18] | 5 | 1.02 [0.91,1.14] | 0.31 | 441,201 | 1278 | 1.10 [1.04,1.16] |
| eGFR** | 774 | 1.07 [1.00, 1.15] | 0.97 | 1.02 [0.94, 1.11] | 34 | 1.07 [1.01, 1.13] | 0.88 | 435,517 | 1270 | 0.79 [.074, 0.85] |
| IGF-1* | 557 | 1.00 [0.99,1.01] | 0.46 | 1.01 [0.99, 1.02] | 9 | 1.01 [1.00,1.02] | 0.44 | 433,371 | 1266 | 1.18 [0.96,1.46] |
| **Lipids** |  |  |  |  |  |  |  |  |  |  |
| Total cholesterol*†* | 726 | 1.06 [1.00,1.12] | 0.58 | 1.07 [0.99, 1.16] | 10 | 1.06 [1.01,1.12] | 0.78 | 435,721 | 1268 | 0.85 [0.80, 0.90] |
| HDL*†* | 835 | 0.91 [0.86,0.97] | <0.01 | 1.01 [0.94, 1.09] | 22 | 0.93 [0.88,0.98] | <0.01 | 398,884 | 1141 | 0.72 [0.66, 0.77] |
| *Small*** | 39 | 1.01 [0.92,1.11] | 0.44 | 0.98 [0.87, 1.10] | 0 | 1.01 [0.92,1.11] | 0.44 | 254,746 | 730 | 0.83 [0.77, 0.89] |
| *Medium*** | 91 | 1.04 [0.97,1.12] | 0.74 | 1.04 [0.95, 1.14] | 1 | 1.05 [0.98,1.13] | 0.60 | 254,746 | 730 | 0.77 [0.71, 0.83] |
| *Large*** | 107 | 1.00 [0.94,1.07] | 0.33 | 1.02 [0.95, 1.10] | 0 | 1.00 [0.94,1.07] | 0.33 | 254,746 | 730 | 0.77 [0.71, 0.83] |
| *Very large*** | 95 | 1.01 [0.95,1.07] | 0.86 | 1.04 [0.96, 1.12] | 1 | 1.01 [0.95,1.07] | 0.84 | 254,746 | 730 | 0.84 [0.77, 0.91] |
| LDL*†* | 559 | 1.00 [0.95,1.06] | 0.69 | 1.01 [0.93, 1.10] | 7 | 1.01 [0.96,1.06] | 0.88 | 434,910 | 1268 | 0.89 [0.84, 0.94] |
| Triglycerides* | 668 | 1.14 [1.07,1.22] | 0.06 | 1.09 [1.00, 1.19] | 16 | 1.11 [1.05,1.17] | 0.09 | 435,386 | 1269 | 1.23 [1.11,1.38] |
| **Sex-hormones** |  |  |  |  |  |  |  |  |  |  |
| SHBG* | 711 | 0.78 [0.69,0.90] | 0.04 | 0.90 [0.75, 1.07] | 18 | 0.80 [0.70,0.90] | 0.04 | 395,122 | 1135 | 0.67 [0.58, 0.76] |
| *Male** | 376 | 0.84 [0.72,0.97] | 0.25 | 0.86 [0.73, 1.01] | 11 | 0.84 [0.75,0.95] | 0.34 | 184,306 | 737 | 0.68 [0.57, 0.82] |
| *Female*** | 351 | 0.82 [0.72,0.93] | 0.44 | 0.83 [0.71, 0.97] | 12 | 0.83 [0.75,0.93] | 0.42 | 210,816 | 398 | 0.79 [0.72, 0.88] |
| Total testosterone |  |  |  |  |  |  |  |  |  |  |
| *Male*** | 237 | 0.94 [0.87,1.01] | 0.96 | 0.93 [0.84, 1.02] | 8 | 0.95 [0.90,1.02] | 0.77 | 199,354 | 806 | 0.85 [0.80, 0.92] |
| *Female** | 286 | 0.96 [0.90,1.03] | 0.18 | 0.94 [0.86, 1.04] | 7 | 0.96 [0.90,1.02] | 0.26 | 196,072 | 370 | 1.22 [0.98, 1.51] |

Adjustments for the cox models: Age, sex, center, education, alcohol status and smoking status, **⬧** SNPs removed by MR-PRESSO and steiger filtering, *log-transformed, **inverse-normal transformation of rank and Z-score, ***Z-score transformation, †standardized and 1-unit increase in Estradiol. Fasting insulin association with RCC was assess in NSHDS.ª Fasting insulin effect as OR [95% CI]. HbA1c: glycated hemoglobin. IGF-1: Insulin-like growth factor-1. eGFR: estimated glomerular filtration rate. HDL/LDL: High/Low-density lipoproteins. SHBG: Sex-hormone binding globulin.

## Table I. Association of potential obesity-related risk factors with risk of clear cell renal cell carcinoma using Mendelian randomization and prospective cohort analyses.

|  | **Mendelian Randomization (MR)** | | | | | | | **Prospective cohort analyses** | | |
| --- | --- | --- | --- | --- | --- | --- | --- | --- | --- | --- |
| **Risk factor** | **SNPs** | **OR (95%CI) IVW** | **MR-Egger intercept (p-value)** | **OR Weighted median** | **SNPs removed⬧** | **Corrected OR (95%CI) IVW** | **Corrected MR-Egger intercept**  **(p-value)** | **N** | **N cases** | **HR/OR (95%CI)** |
| BMI† | 956 | 1.58 [1.46, 1.72] | 0.06 | 1.48 [1.32, 1.66] | 10 | 1.56 [1.44, 1.68] | 0.10 | 461,156 | 1228 | 1.33 [1.27, 1.40] |
| Fasting insulin*ª* | 12 | 1.79 [0.83,3.87] | 0.30 | 3.45 [1.68, 7.09] | 1 | 2.42 [1.23, 4.76] | 0.42 | 378 | 189 | 1.43 [1.02, 2.00] |
| Glucose* | 90 | 0.35 [0.13, 0.94] | 0.76 | 0.38 [0.12, 1.25] | 1 | 0.40 [0.16, 1.01] | 0.90 | 398,602 | 1046 | 1.61 [1.20, 2.17] |
| HbA1c* | 331 | 1.04 [0.56, 1.92] | 0.82 | 0.96 [0.43, 2.14] | 4 | 1.15 [0.64, 2.05] | 0.90 | 432,802 | 1161 | 1.82 [1.29, 2.55] |
| Diastolic blood pressure*** | 259 | 1.18 [1.04,1.34] | 0.45 | 1.14 [0.97, 1.33] | 6 | 1.10 [0.99, 1.23] | 0.76 | 436,517 | 1158 | 1.10 [1.03, 1.16] |
| Systolic blood pressure*** | 223 | 1.04 [0.91,1.20] | 0.60 | 1.00 [0.83, 1.19] | 2 | 1.01 [0.89, 1.15] | 0.19 | 441,201 | 1165 | 1.09 [1.03, 1.16] |
| eGFR** | 756 | 1.08 [0.99, 1.17] | 0.81 | 0.99 [0.90, 1.10] | 26 | 1.04 [0.97, 1.11] | 0.61 | 435,517 | 1163 | 0.79 [0.74, 0.85] |
| IGF-1* | 552 | 1.00 [0.99, 1.01] | 0.58 | 1.01 [1.00, 1.03] | 7 | 1.01 [0.99, 1.02] | 0.53 | 433,371 | 1160 | 1.19 [0.96, 1.48] |
| **Lipids** |  |  |  |  |  |  |  |  |  |  |
| Total cholesterol*†* | 720 | 1.09 [1.02,1.17] | 0.48 | 1.01 [0.92, 1.12] | 7 | 1.10 [1.03,1.17] | 0.69 | 435,721 | 1161 | 0.84 [0.79, 0.89] |
| HDL*†* | 830 | 0.87 [0.81,0.93] | <0.01 | 0.97 [0.88, 1.07] | 17 | 0.89 [0.83,0.94] | <0.01 | 398,884 | 1046 | 0.70 [0.65, 0.76] |
| *Small*** | 37 | 1.02 [0.91,1.15] | 0.92 | 0.93 [0.80, 1.07] | 0 | 1.02 [0.91,1.15] | 0.92 | 254,746 | 668 | 0.84 [0.77, 0.90] |
| *Medium*** | 90 | 1.02 [0.94,1.11] | 0.78 | 1.05 [0.92, 1.19] | 1 | 1.04 [0.96,1.12] | 0.60 | 254,746 | 668 | 0.77 [0.71, 0.84] |
| *Large*** | 104 | 0.99 [0.92,1.08] | 0.25 | 1.03 [0.93, 1.14] | 2 | 0.99 [0.92,1.08] | 0.10 | 254,746 | 668 | 0.76 [0.70, 0.83] |
| *Very large*** | 93 | 1.01 [0.94,1.09] | 0.82 | 1.02 [0.93, 1.12] | 2 | 1.01 [0.95,1.09] | 0.71 | 254,746 | 668 | 0.83 [0.77, 0.91] |
| LDL*†* | 557 | 1.02 [0.96,1.10] | 0.32 | 1.08 [0.97, 1.19] | 5 | 1.01 [0.95,1.08] | 0.64 | 434,910 | 1162 | 0.88 [0.83, 0.94] |
| Triglycerides* | 665 | 1.22 [1.13,1.33] | 0.01 | 1.16 [1.04, 1.30] | 12 | 1.18 [1.10,1.27] | 0.02 | 435,386 | 1163 | 1.22 [1.09, 1.37] |
| **Sex-hormones** |  |  |  |  |  |  |  |  |  |  |
| SHBG* | 691 | 0.73 [0.62,0.86] | 0.13 | 0.82 [0.66, 1.02] | 18 | 0.75 [0.65,0.86] | 0.06 | 395,122 | 1039 | 0.65 [0.57, 0.75] |
| *Male** | 364 | 0.78 [0.65,0.94] | 0.23 | 0.85 [0.69, 1.05] | 15 | 0.83 [0.72,0.96] | 0.43 | 184,306 | 671 | 0.69 [0.57, 0.83] |
| *Female*** | 349 | 0.78 [0.66,0.91] | 0.47 | 0.75 [0.62, 0.92] | 12 | 0.79 [0.69,0.91] | 0.38 | 210,816 | 368 | 0.77 [0.70, 0.86] |
| Total testosterone |  |  |  |  |  |  |  |  |  |  |
| *Male*** | 231 | 0.91 [0.83,1.01] | 0.75 | 0.93 [0.82, 1.06] | 8 | 0.94 [0.87,1.02] | 0.85 | 199,354 | 737 | 0.85 [0.79, 0.92] |
| *Female** | 281 | 0.94 [0.86,1.02] | 0.54 | 0.91 [0.81, 1.03] | 6 | 0.94 [0.86,1.02] | 0.98 | 196,072 | 338 | 1.23 [0.98, 1.55] |

*A*djustments for the cox models: Age, sex, center, education, alcohol status and smoking status, **⬧** SNPs removed by MR-PRESSO and steiger filtering, *log-transformed, **inverse-normal transformation of rank and Z-score, ***Z-score transformation, †standardized and 1-unit increase in Estradiol. Fasting insulin association with RCC was assess in NSHDS. ª Fasting insulin effect as OR [95% CI]. HbA1c: glycated hemoglobin. IGF-1: Insulin-like growth factor-1. eGFR: estimated glomerular filtration rate. HDL/LDL: High/Low-density lipoproteins. SHBG: Sex-hormone binding globulin.

## Table J. Association of potential obesity-related risk factors with risk of papillary renal cell carcinoma using Mendelian randomization and prospective cohort analyses.

|  | **Mendelian Randomization (MR)** | | | | | | | **Prospective cohort analyses** | | |
| --- | --- | --- | --- | --- | --- | --- | --- | --- | --- | --- |
| **Risk factor** | **SNPs** | **OR (95%CI) IVW** | **MR-Egger intercept**  **(p-value)** | **OR Weighted median** | **SNPs removed⬧** | **Corrected OR (95%CI) IVW** | **Corrected MR-Egger intercept**  **(p-value)** | **N** | **N cases** | **HR/OR (95%CI)** |
| BMI† | 954 | 1.29 [1.09, 1.52] | 0.41 | 1.19 [0.93, 1.53] | 1 | 1.28 [1.08, 1.51] | 0.33 | 461,156 | 76 | 1.06 [0.83,1.35] |
| Fasting insulin*ª* | 12 | 0.54 [0.15, 1.92] | 0.07 | 0.42 [0.08, 2.06] | 0 | 0.54 [0.15, 1.92] | 0.07 | - | - | - |
| Glucose* | 90 | 0.54 [0.08, 3.69] | 0.44 | 0.95 [0.06, 14.7] | 0 | 0.54 [0.08, 3.69] | 0.44 | 398,602 | 65 | 0.93 [0.25, 3.49] |
| HbA1c* | 329 | 0.73 [0.24, 2.20] | 0.52 | 0.54 [0.09, 3.28] | 1 | 0.74 [0.24, 2.22] | 0.53 | 432,802 | 74 | 2.37 [0.68, 8.35] |
| Diastolic blood pressure*** | 259 | 1.39 [1.05, 1.83] | 0.92 | 1.14 [0.78, 1.67] | 1 | 1.34 [1.02, 1.74] | 0.85 | 436,517 | 69 | 0.89 [0.70, 1.14] |
| Systolic blood pressure*** | 223 | 1.08 [0.80, 1.47] | 0.83 | 1.14 [0.76, 1.72] | 1 | 1.05 [0.79, 1.40] | 0.44 | 441,201 | 72 | 0.95 [0.74, 1.21] |
| eGFR** | 755 | 1.16 [1.01, 1.33] | 0.39 | 1.24 [0.99, 1.54] | 7 | 1.18 [1.03, 1.34] | 0.70 | 435,517 | 74 | 0.60 [0.47, 0.78] |
| IGF-1* | 552 | 1.01 [0.99, 1.04] | 0.74 | 1.03 [0.99, 1.08] | 0 | 1.01 [0.99, 1.04] | 0.74 | 433,371 | 74 | 1.16 [0.49, 2.77] |
| **Lipids** |  |  |  |  |  |  |  |  |  |  |
| Total cholesterol*†* | 720 | 1.10 [0.96, 1.25] | 0.82 | 0.97 [0.75, 1.26] | 2 | 1.10 [0.97, 1.25] | 0.87 | 435,721 | 74 | 0.80 [0.63, 1.01] |
| HDL*†* | 830 | 0.89 [0.79, 1.01] | 0.14 | 0.87 [0.70, 1.08] | 2 | 0.90 [0.79, 1.02] | 0.13 | 398,884 | 65 | 1.13 [0.86, 1.49] |
| *Small*** | 37 | 1.08 [0.84, 1.38] | 0.11 | 1.48 [1.04, 2.10] | 0 | 1.08 [0.84, 1.38] | 0.11 | 254,746 | 43 | 0.81 [0.60, 1.10] |
| *Medium*** | 90 | 1.02 [0.86, 1.20] | 0.70 | 0.94 [0.69, 1.29] | 0 | 1.02 [0.86, 1.20] | 0.70 | 254,746 | 43 | 0.95 [0.69, 1.32] |
| *Large*** | 104 | 0.98 [0.85, 1.13] | 0.71 | 1.01 [0.79, 1.29] | 0 | 0.98 [0.85, 1.13] | 0.71 | 254,746 | 43 | 1.16 [0.83, 1.63] |
| *Very large*** | 93 | 0.97 [0.85, 1.11] | 0.57 | 0.92 [0.73, 1.16] | 0 | 0.97 [0.85, 1.11] | 0.57 | 254,746 | 43 | 1.08 [0.77, 1.50] |
| LDL*†* | 557 | 1.01 [0.88, 1.16] | 0.58 | 1.11 [0.89, 1.40] | 1 | 1.01 [0.88, 1.16] | 0.65 | 434,910 | 74 | 0.83 [0.66, 1.04] |
| Triglycerides* | 665 | 1.22 [1.05, 1.41] | 0.05 | 1.09 [0.85, 1.40] | 2 | 1.22 [1.06, 1.41] | 0.05 | 435,386 | 74 | 0.58 [0.36, 0.92] |
| **Sex-hormones** |  |  |  |  |  |  |  |  |  |  |
| SHBG* | 687 | 0.90 [0.68, 1.20] | 0.01 | 1.17 [0.73, 1.86] | 3 | 0.95 [0.72, 1.25] | 0.02 | 395,122 | 65 | 1.58 [0.86, 2.90] |
| *Male** | 362 | 0.96 [0.70, 1.32] | 0.01 | 1.09 [0.68, 1.74] | 5 | 1.05 [0.79, 1.41] | 0.05 | 184,306 | 55 | 1.59 [0.79, 3.17] |
| *Female*** | 348 | 1.08 [0.82, 1.41] | 0.45 | 1.32 [0.84, 2.08] | 0 | 1.08 [0.82, 1.41] | 0.45 | 210,816 | 10 | 1.22 [0.65, 2.28] |
| Total testosterone |  |  |  |  |  |  |  |  |  |  |
| *Male*** | 230 | 0.95 [0.80, 1.12] | 0.07 | 0.85 [0.66, 1.10] | 0 | 0.95 [0.80, 1.12] | 0.07 | 199,354 | 57 | 0.93 [0.72, 1.21] |
| *Female** | 281 | 0.96 [0.82, 1.13] | 0.88 | 0.99 [0.76, 1.29] | 1 | 0.96 [0.81, 1.12] | 0.88 | 196,072 | 10 | 0.68 [0.18, 2.63] |

Adjustments for the cox models: Age, sex, center, education, alcohol status and smoking status, **⬧** SNPs removed by MR-PRESSO and steiger filtering, *log-transformed, **inverse-normal transformation of rank and Z-score, ***Z-score transformation, †standardized and 1-unit increase in Estradiol. Fasting insulin association with RCC was assess in NSHDS. ª Fasting insulin effect as OR [95% CI]. HbA1c: glycated hemoglobin. IGF-1: Insulin-like growth factor-1. eGFR: estimated glomerular filtration rate. HDL/LDL: High/Low-density lipoproteins. SHBG: Sex-hormone binding globulin.

## Table K. Comparison between the adjusted and the non-adjusted model for specific female factors.

| **Outcome** | **Risk factor** | **Model non adjusted for female factor*** | **Pvalue** | **Model adjusted for female factors**** | **Pvalue** |
| --- | --- | --- | --- | --- | --- |
| **RCC** | SHBG females | 0.79 [0.72, 0.88] | <0.001 | 0.79 [0.71, 0.87] | <0.001 |
| **RCC** | Testosterone females | 1.22 [0.98, 1.51] | 0.08 | 1.25 [1.00, 1.55] | 0.05 |
| **ccRCC** | SHBG females | 0.77 [0.70, 0.86] | <0.001 | 0.76 [0.69, 0.85] | <0.001 |
| **ccRCC** | Testosterone females | 1.23 [0.98, 1.55] | 0.07 | 1.27 [1.01, 1.60] | 0.04 |

*Cox proportional hazard model adjusted for age, center of recruitment, education, smoking status and alcohol drinking status. ** Cox proportional hazard model additionally adjusted for Hormone replacement therapy (Yes/no) and menopausal status (Yes/no). RCC: renal cell carcinoma. ccRCC: clear cell renal cell carcinoma. SHBG: Sex-hormone binding globulin.

## Table L. Beta estimates between potential mediators

| **Mediator 1 - Mediator 2** | **Mediator 1 -> Mediator 2**  **Beta (pvalue)** | | **Mediator 2 -> Mediator 1**  **Beta (pvalue)** | |
| --- | --- | --- | --- | --- |
|  | **Mendelian randomization** | **Cohort** | **Mendelian randomization** | **Cohort** |
| Insulin - Diastolic BP | 0.20 (0.006) | - | Pvalue >0.05 | - |
| Insulin - HDL | -0.50 (0.007) | - | -0.05 (<0.001) | - |
| Insulin - Triglycerides | 0.48 (0.01) | - | 0.05 (<0.001) | - |
| Insulin - SHBG overall | -0.22 (0.016) | - | -0.12 (0.001) | - |
| Insulin - SHBG males | -0.18 (0.024) | - | -0.08 (0.003) | - |
| Insulin - SHBG females | -0.27 (0.009) | - | -0.12 (<0.001) | - |
|  |  |  |  |  |
| Diastolic BP - HDL | -0.07 (<0.001) | -0.02 (<0.001) | Pvalue >0.05 | -0.03 (<0.001) |
| Diastolic BP - Triglycerides | 0.07 (<0.001) | 0.08 (<0.001) | 0.05 (0.006) | 0.31 (<0.001) |
| Diastolic BP – SHBG overall | -0.02 (0.009) | -0.08 (<0.001) | -0.09 (<0.001) | -0.35 (<0.001) |
| Diastolic BP - SHBG males | -0.02 (0.047) | -0.05 (<0.001) | -0.30 (<0.001) | -0.30 (<0.001) |
| Diastolic BP - SHBG females | -0.03 (0.002) | -0.19 (<0.001) | -0.07 (0.002) | -0.18 (<0.001) |
|  |  |  |  |  |
| HDL - Triglycerides | -0.29 (<0.001) | -0.25 (<0.001) | -0.53 (<0.001) | -0.80 (<0.001) |
| HDL – SHBG overall | 0.04 (<0.001) | 0.16 (<0.001) | 0.31 (<0.001) | 0.62 (<0.001) |
| HDL - SHBG males | 0.03 (<0.001) | 0.13 (<0.001) | 0.24 (<0.001) | 0.54 (<0.001) |
| HDL - SHBG females | 0.05 (<0.001) | 0.37 (<0.001) | 0.29 (<0.001) | 0.35 (<0.001) |
|  |  |  |  |  |
| Triglycerides – SHBG overall | -0.07 (<0.001) | -0.34 (<0.001) | -0.36 (<0.001) | -0.40 (<0.001) |
| Triglycerides - SHBG males | -0.06 (<0.001) | -0.28 (<0.001) | -0.28 (<0.001) | -0.48 (<0.001) |
| Triglycerides - SHBG females | -0.06 (<0.001) | -0.76 (<0.001) | -0.30 (<0.001) | -0.17 (<0.001) |

Association between each potential mediator, bidirectionally, in cohort and MR analyses. BP: blood pressure. SHBG: Sex-hormone binding globulin. HDL: High-density lipoprotein cholesterol. Each model, in the cohort analyses, was adjusted for age, sex, center of recruitment, education, alcohol and smoking status.

## Table M. Proportion of BMI effect on renal cell carcinoma mediated.

| **Mediator** | **Mendelian Randomization (MR)** | | | **Prospective cohort analyses** | | |
| --- | --- | --- | --- | --- | --- | --- |
|  | **Total effect**  **OR [95% CI]** | **Indirect effect OR [95% CI]** | **Proportion mediated** | **Total effect**  **HR/OR [95% CI]** | **Indirect effect**  **HR [95% CI]** | **Proportion mediated** |
| Fasting Insulinª | 1.44 [1.35, 1.54] | 1.09 [0.97, 1.21] | 22.4 % | 1.18 [0.96, 1.46] | 1.12 [1.04, 1.20] | 66.6 % |
| Diastolic BP |  | 1.04 [0.99, 1.09] | 11.0 % | 1.33 [1.26, 1.39] | 1.01 [0.99, 1.03] | 3.64 % |
| HDL |  | 1.02 [0.99, 1.05] | 4.55 % |  | 1.08 [1.06, 1.10] | 27.4 % |
| Triglycerides |  | 1.03 [0.99, 1.08] | 8.94 % |  | 1.01 [0.98, 1.04] | 3.26 % |
| SHBG |  | 1.04 [0.96, 1.12] | 10.0 % |  | 1.04 [1.00, 1.08] | 13.5 % |
| *Male* |  | 1.01 [0.94, 1.09] | 2.38 % |  | 1.03 [0.97, 1.08] | 9.44 % |
| *Female* |  | 1.05 [0.98, 1.13] | 14.5 % |  | 1.06 [1.02, 1.09] | 19.5 % |

Each cox proportional hazard model was adjusted for age, sex, center of recruitment, education, smoking and alcohol drinking status. ªFasting insulin effect as OR [95% CI]. BP: blood pressure. SHBG: Sex-hormone binding globulin. HDL: High-density lipoprotein cholesterol.

## Table N. Proportion of BMI effect on ccRCC mediated.

| **Mediator** | **Mendelian Randomization (MR)** | | | **Prospective cohort analyses** | | |
| --- | --- | --- | --- | --- | --- | --- |
|  | **Total effect**  **OR [95% CI]** | **Indirect effect**  **OR [95% CI]** | **Proportion mediated** | **Total effect**  **HR/OR [95% CI]** | **Indirect effect**  **HR [95% CI]** | **Proportion mediated** |
| Fasting insulinª | 1.56 [1.44, 1.68] | 1.10 [0.93, 1.31] | 22.3 % | 1.18 [0.96, 1.46] | 1.12 [1.04, 1.20] | 66.6 % |
| HDL |  | 1.04 [0.99, 1.08] | 7.96 % | 1.33 [1.27, 1.40] | 1.09 [1.06, 1.11] | 28.7 % |
| Triglycerides |  | 1.05 [0.99, 1.11] | 10.2 % |  | 1.01 [0.97, 1.04] | 2.16 % |
| SHBG |  | 1.03 [0.92, 1.16] | 7.21 % |  | 1.04 [1.00, 1.09] | 13.9 % |
| *Male* |  | 1.01 [0.90, 1.12] | 1.34 % |  | 1.03 [0.97, 1.09] | 8.90 % |
| *Female* |  | 1.05 [0.95, 1.16] | 10.7 % |  | 1.07 [1.03, 1.10] | 21.9 % |

Each cox proportional hazard model was adjusted for age, sex, center of recruitment, education, smoking and alcohol drinking status. ªFasting insulin effect as OR [95% CI]. SHBG: Sex-hormone binding globulin. HDL: High-density lipoprotein cholesterol.

## Table O. Multivariable analyses of mediators on renal cell carcinomas.

|  | **Univariate analysis**  **Mendelian Randomization** | **Univariate analysis Prospective cohort analyses** | **Mediator** | **Multivariable**  **Mendelian Randomization** | **Multivariable**  **Prospective cohort analyses** |
| --- | --- | --- | --- | --- | --- |
| **Diastolic BP** | 1.14 [1.04, 1.26] | 1.11 [1.05, 1.17] | HDL Cholesterol | - | 1.10 [1.03, 1.17] |
|  |  |  | Triglycerides | - | 1.10 [1.04, 1.16] |
|  |  |  | SHBG *overall* | - | 1.07 [1.01, 1.14] |
|  |  |  | SHBG *males* | - | 1.06 [0.99, 1.15] |
|  |  |  | SHBG *females* | - | 1.09 [0.98, 1.21] |
| **HDL Cholesterol** | 0.93 [0.88, 0.98] | 0.72 [0.66, 0.77] | Diastolic BP | 0.76 [0.54, 1.08] | 0.71 [0.66, 0.76] |
|  |  |  | Triglycerides | 1.00 [0.93, 1.08] | 0.70 [0.65, 0.76] |
|  |  |  | SHBG *overall* | 0.86 [0.76, 0.99] | 0.75 [0.69, 0.81] |
|  |  |  | SHBG *males* | 0.91 [0.76, 1.09] | 0.73 [0.66, 0.81] |
|  |  |  | SHBG *females* | 0.87 [0.74, 1.02] | 0.77 [0.69, 0.87] |
| **Triglycerides** | 1.11 [1.05, 1.17] | 1.23 [1.11, 1.38] | Diastolic BP | 2.00 [1.40, 2.87] | 1.20 [1.08, 1.35] |
|  |  |  | HDL Cholesterol | 1.16 [1.06, 1.27] | 0.95 [0.84, 1.08] |
|  |  |  | SHBG *overall* | 1.13 [1.02, 1.25] | 1.08 [0.95, 1.22] |
|  |  |  | SHBG *males* | 1.13 [1.01, 1.26] | 0.98 [0.85, 1.14] |
|  |  |  | SHBG *females* | 1.17 [1.02, 1.34] | 1.36 [1.09, 1.69] |
| **SHBG overall** | 0.80 [0.72, 0.90] | 0.67 [0.58, 0.76] | Diastolic BP | 0.77 [0.52, 1.13] | 0.68 [0.60, 0.78] |
|  |  |  | HDL Cholesterol | 0.67 [0.55, 0.81] | 0.77 [0.67, 0.89] |
|  |  |  | Triglycerides | 0.82 [0.69, 0.97] | 0.68 [0.59, 0.78] |
| **SHBG females** | 0.83 [0.75, 0.93] | 0.79 [0.72, 0.88] | Diastolic BP | 0.86 [0.62, 1.19] | 0.80 [0.72, 0.89] |
|  |  |  | HDL Cholesterol | 0.72 [0.60, 0.86] | 0.86 [0.77, 0.96] |
|  |  |  | Triglycerides | 0.82 [0.70, 0.95] | 0.84 [0.75, 0.93] |

Multivariable analyses between each potential mediator adjusted for each other mediator. Each cox proportional hazard model was adjusted for age, sex, center of recruitment, education, smoking and alcohol drinking status. Not enough SNPs in Insulin instruments to run multivariable MR with another mediator. - Not calculated to due lack of power (F-statistic_conditional_ <10). BP: blood pressure. SHBG: Sex-hormone binding globulin. HDL: High-density lipoprotein.

**References**

1. Yengo L, Sidorenko J, Kemper KE, Zheng Z, Wood AR, Weedon MN, et al. Meta-analysis of genome-wide association studies for height and body mass index in ∼700000 individuals of European ancestry. Hum Mol Genet. 2018;27: 3641. doi:10.1093/HMG/DDY271

2. Lagou V, Mägi R, Hottenga JJ, Grallert H, Perry JRB, Bouatia-Naji N, et al. Sex-dimorphic genetic effects and novel loci for fasting glucose and insulin variability. Nature Communications 2021 12:1. 2021;12: 1–18. doi:10.1038/s41467-020-19366-9

3. Johansson M, Carreras-Torres R, Scelo G, Purdue MP, Mariosa D, Muller DC, et al. The influence of obesity-related factors in the etiology of renal cell carcinoma—A mendelian randomization study. PLoS Med. 2019;16: e1002724. doi:10.1371/JOURNAL.PMED.1002724

4. Karczewski KJ, Gupta R, Kanai M, Lu W, Tsuo K, Wang Y, et al. Pan-UK Biobank genome-wide association analyses enhance discovery and resolution of ancestry-enriched effects. Nat Genet. 2025;57. doi:10.1038/S41588-025-02335-7

5. Sinnott-Armstrong N, Tanigawa Y, Amar D, Mars N, Benner C, Aguirre M, et al. Genetics of 35 blood and urine biomarkers in the UK Biobank. Nat Genet. 2021;53: 185. doi:10.1038/S41588-020-00757-Z

6. Graham SE, Clarke SL, Wu KHH, Kanoni S, Zajac GJM, Ramdas S, et al. The power of genetic diversity in genome-wide association studies of lipids. Nature. 2021;600: 675. doi:10.1038/S41586-021-04064-3

7. Davyson E, Shen X, Gadd DA, Bernabeu E, Hillary RF, McCartney DL, et al. Metabolomic investigation of major depressive disorder identifies a potentially causal association with polyunsaturated fatty acids. Biol Psychiatry. 2023;94: 630. doi:10.1016/J.BIOPSYCH.2023.01.027

8. Ruth KS, Day FR, Tyrrell J, Thompson DJ, Wood AR, Mahajan A, et al. Using human genetics to understand the disease impacts of testosterone in men and women. Nature Medicine 2020 26:2. 2020;26: 252–258. doi:10.1038/s41591-020-0751-5
