# Supplementary material for: Systematic assessment of obesity-related risk factors in renal cancer etiology: A longitudinal risk and Mendelian randomization analysis
Source: PLoS Med. 2026 Feb 10;23(2):e1004906. doi: 10.1371/journal.pmed.1004906 (PMC12919923; doi:10.1371/journal.pmed.1004906)
Supplement: S1 Appendix — (DOCX) [file pmed.1004906.s006.docx]

Alcala, Mariosa, Jacobson, Coscia-Requena, Dimou, Franklin, Martin, Davey Smith, Gunter, Brennan, Pollak, Langdon, Johansson. Systematic assessment of obesity-related risk factors in renal cancer etiology: A longitudinal risk and Mendelian randomization analysis

Contents

[Abbreviations 1](#_Toc214284513)

[Supplementary methods & materials 2](#_Toc214284514)

[1. Study population, UK Biobank 2](#_Toc214284515)

[2. Fasting insulin measurements 2](#_Toc214284516)

[3. Genome-wide association studies 2](#_Toc214284517)

[4. Mendelian Randomization (MR) 3](#_Toc214284518)

# Abbreviations

BMI: Body mass index

DBP: Diastolic blood pressure

eGFR: Estimated glomerular filtration rate

GWAS: Genome wide association study

HbA1c: Glycated hemoglobin

HDL: High-density lipoprotein

LDL: Low-density lipoprotein

MR: Mendelian Randomization

NSHDS: The Northern Sweden Health and Disease Study

RCC: Renal cell carcinoma

SBP: Systolic blood pressure

SHBG: Sex-hormone binding globulin

SNP: Single nucleotide polymorphism

UKB: UK Biobank

# Supplementary methods & materials

## Study population, UK Biobank

In addition to kidney cancer (ICD10 code: C64), we evaluated two subtypes as outcomes: clear cell renal cell carcinoma (ccRCC, histology codes: 8050, 8140, 8260, 8270, 8280, 8290, 8310, 8312, 8316-8320, 8340-8344) and papillary renal cell carcinoma (pRCC, 8260).

Diastolic blood pressure (DBP, mmHg) and systolic blood pressure (SBP, mmHg) were taken twice, few minutes in between, by an automated reading at enrolment. We used the mean of these two measurements, except if the difference was greater than 10 for DBP and 20 for SBP, in that case it was set as missing.

Analyses were performed in complete data, without imputation, we excluded participants with missing BMI (<1%), missing biomarker measurements of which most (80%) occurred due to a missing blood sample or a combination of technical issues, and markers (HDL particles and total cholesterol) quantified by nuclear magnetic resonance (NMR) were performed for 275,240 participants, missing data among them were due to technical issues.

## Fasting insulin measurements

Blood samples were collected in natrium-heparin and EDTA-tubes, centrifuged, aliquoted, and frozen in -25 °C within an hour. After transportation to the biobank within a week, the samples were stored in -80 °C. Included case and control plasma samples were organized pairwise in random order, and the analyses were performed by technicians blinded to case-control status. Insulin levels were measured in a specialized lab at McGill University in Canada using sandwich-type ELISA methods (Mercodia Insulin ELISA (Mercodia AB, Uppsala, Sweden)).

## Genome-wide association studies

We performed GWAS for glucose and glycated haemoglobin (HbA1c) measurements. We used UK Biobank measurements to have the same fasting status than in the prospective cohort analysis. Glucose and HbA1c were log transformed and GWAS adjusted for age, sex and the first ten principal components.

## Mendelian Randomization (MR)

Valid instruments for MR are defined by three core assumptions:[1]

- Relevance assumption: the genetic variants are associated with the exposure.
- Independence assumption: the genetic variants are not associated with any confounding factors that bias the association between the genetic instruments and the outcome.
- Exclusion restriction assumption: the genetic variants do not affect the outcome except through the given risk factor.

To comply with these assumptions, for each set of genetic instruments, we excluded non-genome-wide significant SNPS (p-value >5.10^-8^), SNPs with a low imputation quality (<0.3) and SNPs with a minor allele frequency less than 0.01. We additionally excluded correlated SNPs in linkage disequilibrium (r2>0.01 and separated by less than 10 000 kb). SNPs available for mediators but not for outcome and palindromic SNPs, if the minor allele frequency was below 0.42, were replaced, if possible, by proxy SNPs in genetic linkage (r2>0.8) based on the Ensembl Project [2].

We estimated the strength of the relationship between each instrument and the outcome using the F-statistics (F-statistics<10 denote weak instruments), the lower F-statistics was 43 for Insulin and the higher was 149 for LDL cholesterol.

In addition, we evaluated the robustness of our results by assessing the presence of horizontal pleiotropy using the MR-Egger method[3], and compared the weighted median estimate to the IVW to test for the weak instruments biases. We used MR-PRESSO method to locate and exclude outliers SNPs [4]. In addition, we applied a Steiger filtering[5], between each exposure and outcome, to remove the SNPs more associated with the outcome than the exposure.

In the multivariable MR, after verification of the unbalanced pleiotropy by the MR-Egger method and removing the outliers identified by the MR-PRESSO we applied a Steiger filtering. As it is not a standard developed method, we first identified SNPs to be removed from the BMI to RCC couple (step 1), then SNPs from the mediator- RCC couple (step 2) and finally SNPs from the BMI-RCC couple (step 3). We removed SNPs dropped in step 3 from step 1 (step 4), then kept all SNPs selected in step 2 and 4. We then selected all these SNPs from the exposure, mediator and outcome GWAS, harmonized to ensure that all same effect estimates corresponded to the same effect allele for all instruments, and ran the multivariable MR. To assess the strength of the association between our genetic instruments and the exposure, we performed the F-conditional statistics, the lowest among our selected mediators is from DBP (F-statistic_conditional_ =8), all others shown a F-statistic_conditional_ above 10.

**References**

1. Lawlor DA, Harbord RM, Sterne JAC, Timpson N, Smith GD. Mendelian randomization: Using genes as instruments for making causal inferences in epidemiology. Stat Med. 2008;27: 1133–1163. doi:10.1002/SIM.3034

2. Howe KL, Achuthan P, Allen J, Allen J, Alvarez-Jarreta J, Ridwan Amode M, et al. Ensembl 2021. Nucleic Acids Res. 2021;49: D884–D891. doi:10.1093/nar/gkaa942

3. Bowden J, Davey Smith G, Burgess S. Mendelian randomization with invalid instruments: effect estimation and bias detection through Egger regression. Int J Epidemiol. 2015;44: 512–525. doi:10.1093/ije/dyv080

4. Verbanck M, Chen CY, Neale B, Do R. Detection of widespread horizontal pleiotropy in causal relationships inferred from Mendelian randomization between complex traits and diseases. Nat Genet. 2018;50: 693–698. doi:10.1038/s41588-018-0099-7

5. Hemani G, Tilling K, Davey Smith G. Orienting the causal relationship between imprecisely measured traits using GWAS summary data. PLoS Genet. 2017;13: e1007081. doi:10.1371/JOURNAL.PGEN.1007081
