# Supplementary material for: Systematic assessment of obesity-related risk factors in renal cancer etiology: A longitudinal risk and Mendelian randomization analysis
Source: PLoS Med. 2026 Feb 10;23(2):e1004906. doi: 10.1371/journal.pmed.1004906 (PMC12919923; doi:10.1371/journal.pmed.1004906)
Supplement: Attachment — Submitted filename: Revisions_answers.docx [file pmed.1004906.s009.docx]

Revisions answers

# Editor’s comments:

GENERAL
* Please confirm that your title complies with to PLOS Medicine's style. Your title must be nondeclarative and not a question. It should begin with main concept if possible. "Effect of" should be used only if causality can be inferred, i.e., for an RCT. Please place the study design ("A randomized controlled trial," "A retrospective study," "A modelling study," etc.) in the subtitle (ie, after a colon).

We revised our title according to the PLOS Medicine’s style: Systematic assessment of obesity-related risk factors in renal cancer etiology: An observational study using both population cohorts and genetic studies.

* Statistical reporting: Please revise throughout the manuscript, including tables and figures.
- Please report statistical information as follows to improve clarity for the reader ""22% (95% CI [13,28]; p</=)"".
- Please separate upper and lower bounds with commas instead of hyphens as the latter can be confused with reporting of negative values.
- Please repeat statistical definitions (HR, CI etc.) for each set of parentheses.

All p-value have been added to the main text. We also replaced all hyphens by commas between lower and upper bounds.

* Please ensure that all abbreviations are defined at first use throughout the text (including statistical abbreviations).

We added the complete description for each abbreviation.

* Please ensure that tables and figures, including those in supplementary files, are appropriately referenced in the main text.

We checked all the references to the tables and figures in the main text.

* Please review your text for claims of novelty or primacy (e.g. 'for the first time' or ‘novel’) and remove this language.

We replaced all sentences that could claim of novelty or primacy.

* Please confirm that any use of statistical terms (such as trend or significant) are supported by the data, and if not please remove them. The term trend should be used only when the test for trend has been conducted.

Yes we confirm, these terms were not used in this manuscript.

* Please provide titles and legends for all figures and tables (including those in Supporting Information files).

A title and a legend were added to each figure and table.

* Please define all acronyms used in each figure or table in the corresponding legend.
All acronyms used in figure and tables have been added to the corresponding legend.

* Please confirm that you used patient-centered language. Please note that patient-centered language is constructed with the use of post-modified nouns putting the person first in the sentence structure.

Yes, we confirm.

* Please include the statement on code availability in the data availability statement in the online submission form.

* Please add this statement to the manuscript's Competing Interests: "GDS is an Academic Editor on PLOS Medicine's editorial board."

This statement has been added under the Competing Interests section.

* Thank you for agreeing to make your data available. At this time, please provide the link to the data repository and accession numbers required for access.

The corresponding data repository is available in Gitlab: https://gitlab.com/Karine.Alcala/obesity_rcc/-/tree/79b0a4566da2a0a29f3bbf08eaf1d75f66691f69/ and archived in Zenodo: https://doi.org/10.5281/zenodo.17640162

No accession numbers are required to access the files.

ABSTRACT
* Please confirm that your abstract complies with our requirements, including providing all the information relevant to this study type <https://journals.plos.org/plosmedicine/s/submission-guidelines#loc-abstract>

We added the follow-up information and main limitation in the abstract, otherwise, we confirm that we followed the requirements.

* Please confirm that all numbers presented in the abstract are present and identical to numbers presented in the main manuscript text.

We confirm that all numbers presented in the abstract are also present and identical in the main manuscript text.

* In the abstract, please include the important dependent variables that are adjusted for in the analyses.

We added this information in the abstract.

* In the last sentence of the Abstract Methods and Findings section, please describe the main limitation(s) of the study's methodology.

The main limitation was added at the end of the Methods and findings section in the abstract.

METHODS AND RESULTS
* The terms gender and sex are not interchangeable (as discussed in <https://www.who.int/health-topics/gender#tab=tab_1> ); please use the appropriate term.

We replaced any mention to gender, men or women in the text and replaced by sex, males and females.

* “We selected each set of genetic instruments according to the gold standard for Mendelian Randomization analyses, previously described (Detailed methods in S1 Appendix) [29].” – Please provide a summary of the methods with sufficient detail in the main text.

We added information in the main text regarding the genetic instruments selection process.

* “As a sensitivity analysis, for sex-hormones in females, we adjusted our models in the observational analysis with hormone-replacement therapy and menopausal status which did not alter the results.” – please provide a reference to the relevant table/figure.

Supplementary Table 11 has been added to display these results.

* “When considering individual risk factors as mediators, we estimated that insulin mediates between 67% and 22% of the effect of elevated BMI on renal cancer risk based on the cohort and genetic analyses, respectively (Figure 4).” – please explain where 67% and 22% come from? Are these numbers available outside the figure? If so, please ensure to add a relevant reference.

We rephrased this sentence and added the reference to the corresponding table.

* Please confirm that you specified the variables controlled for in all relevant Tables/Figures.

We added this information in each Table/Figure legend when relevant.

------------------------------------------------------------
General Editorial Requests

1) We ask every co-author listed on the manuscript to fill in a contributing author statement. If any of the co-authors have not filled in the statement, we will remind them to do so when the paper is revised. If all statements are not completed in a timely fashion this could hold up the re-review process. Should there be a problem getting one of your co-authors to fill in a statement we will be in contact. YOU MUST NOT ADD OR REMOVE AUTHORS UNLESS YOU HAVE ALERTED THE EDITOR HANDLING THE MANUSCRIPT TO THE CHANGE AND THEY SPECIFICALLY HAVE AGREED TO IT.

2) Please ensure that the paper adheres to the PLOS Data Availability Policy (see <http://journals.plos.org/plosmedicine/s/data-availability)>, which requires that all data underlying the study's findings be provided in a repository or as Supporting Information. For data residing with a third party, authors are required to provide instructions with contact information for obtaining the data. PLOS journals do not allow statements supported by "data not shown" or "unpublished results." For such statements, authors must provide supporting data or cite public sources that include it.

3) Please review your reference list to ensure that it is complete and correct. If you have cited papers that have been retracted, please include the rationale for doing so in the manuscript text, or remove these references and replace them with relevant current references. Any changes to the reference list should be mentioned in the rebuttal letter that accompanies your revised manuscript.
Reference list has been reviewed.
